# Bandwidth Optimization Design of a Multi Degree of Freedom MEMS Gyroscope

**DOI:** 10.3390/s130810550

**Published:** 2013-08-14

**Authors:** Chaowei Si, Guowei Han, Jin Ning, Fuhua Yang

**Affiliations:** 1 Institute of Semiconductors, Chinese Academy of Science, Beijing 100083, China; E-Mails: schw@semi.ac.cn (C.S.); hangw1984@semi.ac.cn (G.H.); 2 Department of Electronic Engineering, Tsinghua University, Beijing 100084, China

**Keywords:** multi-DOF MEMS gyroscope, robustness, narrow bandwidth, large amplification factor

## Abstract

A new robust multi-degree of freedom (multi-DOF) MEMS gyroscope is presented in this paper. The designed gyroscope has its bandwidth and amplification factor of the sense mode adjusted more easily than the previous reported multi-DOF MEMS gyroscopes. Besides, a novel spring system with very small coupling stiffness is proposed, which helps achieve a narrow bandwidth and a high amplification factor for a 2-DOF vibration system. A multi-DOF gyroscope with the proposed weak spring system is designed, and simulations indicate that when the operating frequency is set at 12.59 kHz, the flat frequency response region of the sense mode can be designed as narrow as 80 Hz, and the amplification factor of the sense mode at the operating frequency is up to 91, which not only protects the amplification factor from instability against process and temperature variations, but also sacrifices less performance. An experiment is also carried out to demonstrate the validity of the design. The multi-DOF gyroscope with the proposed weak coupling spring system is capable of achieving a good tradeoff between robustness and the performance.

## 1. Introduction

Most studies on MEMS gyroscopes are focused on their performance, and common methods to improve the performance include improving quality factor enhancement [1], quadrature error cancellation [2], and mode matching [3], based on which the bias drift has already been driven below 0.1 degrees/h [4,5]. On the other side, the robustness has also been considered, and multi-DOF MEMS gyroscopes are proved to display good robustness and long-term stability, as they are insensitive to structural and environmental parameter variations [6–8].

A series of tests on 3-DOF MEMS gyroscopes show that the amplification factor is insensitive to temperature variation, power supply jitter and damping change [9]. A more robust multi-DOF gyroscope with 2-DOF sense mode and 2-DOF drive mode is further reported [10], but the improvement of the robustness affects the sensitivity.

For the 2-DOF vibration system consisted of two spring mass systems in series described in [6–11], the amplification factor of the 2-DOF vibration mode is inversely proportional to the square of the bandwidth [11], but unfortunately, the bandwidth is hard to narrow enough to get ideal amplification factor, because the bandwidth is determined by the spring stiffness ratio and the mass ratio, and those ratios are within limits due to process capacities.

The paper adopts another 2-DOF vibration system consisted of two spring mass systems connected by a coupling spring, from the equivalent mechanical model, it is found that the increase of the amplification factor relies on the decrease of the coupling spring stiffness. Furthermore, a novel spring system of very small coupling spring stiffness is proposed. Simulations of a reasonably designed gyroscope with the proposed spring system indicate that the amplification factor is up to 91 when the peak to peak bandwidth is narrowed to 240 Hz.

## 2. Work Principle and Design

### 2.1. Analysis of 2-DOF Vibration Systems

Generally, in the fabrication of a MEMS gyroscope, resonant frequency drift caused by the process variations could be up to tens of Hertz [7]. Meanwhile, the resonant frequency separation of the drive mode and the sense mode varies with the operating temperature, both of which influence the performance. The performance loss due to the fabrication errors and the temperature variations can be prevented if the frequency response in the sense mode is flat in a specific range [9], and for all reported multi-DOF gyroscopes [6–11], the bandwidth meets the demand, but a large bandwidth results in a small amplification factor, in other words, the sensitivity of the gyroscope is decreased [11].

The 2-DOF vibration systems mentioned in [6,9] consist of two spring mass systems in series, and the way to enlarge the amplification factor is to make it operate in a low central frequency and decrease the mass ratio and the spring stiffness ratio. To realize a flat region about 200 Hz, the drive mode resonant frequency is designed to be 752 Hz, with a mass ratio of 0.0624 and a spring stiffness ratio of 0.0429 in [9]. Moreover, the mass ratio is set to 0.05 when the central frequency is about 2 kHz in [6]. It is difficult to narrow the bandwidth further for larger mass ratio and spring stiffness ratio requires smaller masses and longer folded beams, which are limited by the current fabrication capabilities.

**Figure 1.**
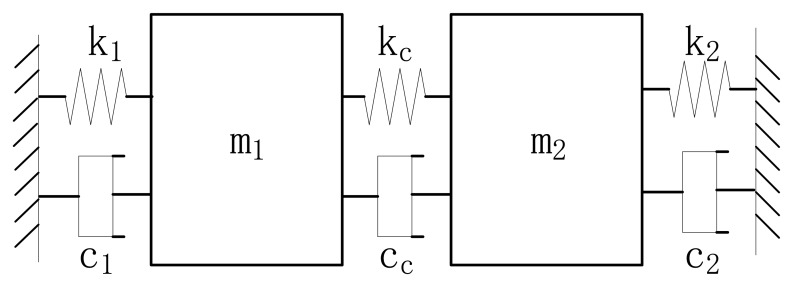
The mechanical model of a 2-DOF vibration system.

It is less complicated to adjust the bandwidth of a 2-DOF vibration system consisting of two spring mass systems connected by a coupling spring, whose equivalent mechanical model is shown in Figure 1.

One of the spring mass systems consists of a support spring *k*_1_, a mass *m*_1_ and the damping factor is *c*_1_, the other is composed of a support spring *k*_2_, a mass *m*_2_ and the damping factor is *c*_2_. The two spring mass systems are connected by a coupling spring *k_c_* with an extra damping factor *c_c_* introduced. The resonant frequency of the first spring mass system added with *k_c_* is *ω_s_*_1_, the resonant frequency the latter added with *k_c_* is *ω_s_*_2_, that means *ω_s_*_1_^2^ = (*k*_1_ + *k_c_*)/*m*_1_, *ω_s_*_2_^2^ = (*k*_2_ + *k*_c_)/*m*_2_. The spring stiffness ratio of *k_c_* to *k*_1_ is *r_c_*_1_, the spring stiffness ratio of *k_c_* to *k*_2_ is *r_c_*_2_, the central frequency is *ω*_0_, and the two peak frequencies of the 2-DOF system are *ω*_1_ and *ω*_2_, which are revealed in Equations (1) and (2):

(1) $${\omega_{1}}^{2}=\frac{{\omega_{s2}}^{2}}{2}+\frac{{\omega_{s1}}^{2}}{2}+\sqrt{{\left(\frac{{\omega_{s2}}^{2}}{2}-\frac{{\omega_{s1}}^{2}}{2}\right)}^{2}+\frac{r_{c1}r_{c2}}{\left(1+r_{c1}\right)\left(1+r_{c2}\right)}{\omega_{s1}}^{2}{\omega_{s2}}^{2}}$$

(2) $${\omega_{2}}^{2}=\frac{{\omega_{s2}}^{2}}{2}+\frac{{\omega_{s1}}^{2}}{2}+\sqrt{{\left(\frac{{\omega_{s2}}^{2}}{2}-\frac{{\omega_{s1}}^{2}}{2}\right)}^{2}+\frac{r_{c1}r_{c2}}{\left(1+r_{c1}\right)\left(1+r_{c2}\right)}{\omega_{s1}}^{2}{\omega_{s2}}^{2}}$$

The peak to peak bandwidth *ω*_1_− *ω*_2_ is defined as *BW_pp_*. Obviously, *BW_pp_* get a minimum when *ω_s_*_1_ = *ω_s_*_2_ = *ω_s_*. Assuming *p*^2^ = *r_c_*_1_*r_c_*_2_/((1 + *r_c_*_1_)(1 + *r_c_*_2_)), *BW_pp_* is derived in Equation (3):

(3) $$BW_{pp}=\omega_{2}-\omega_{1}=\left(\sqrt{1+p}-\sqrt{1-p}\right)\omega_{s}\approx p\omega_{s}$$

By truncating appropriately the Taylor series expansion for $\sqrt{1+p}$ and $\sqrt{1-p}$, *BW_pp_* is acquired in Equation (4):

(4) $$BW_{pp}\approx p\omega_{s}$$

and the central frequency *ω*_0_ is derived in Equation (5):

(5) $$\omega_{0}=\frac{\omega_{2}+\omega_{1}}{2}=\frac{\left(\sqrt{1+p}+\sqrt{1-p}\right)}{2}\omega_{s}$$

Taylor series expansions are used to simplify the above equation, and a simplified equation of *ω*_0_ is given in Equation (6).

(6) $$\omega_{0}\approx \omega_{s}$$

From Equation (4), *BW_pp_* is proportional to *p* when *ω_s_* is given, so the bandwidth optimization is simplified as there is only one parameter to adjust.

In a MEMS gyroscope, a spring is often made up of a number of folded beams in series, and the stiffness of the spring is inversely proportional to the total effective length. According to the above analyses, narrow bandwidth requires a coupling spring of very small stiffness, and if *k_c_* is made up of traditional folded beams, it should have a length tens of the support spring to achieve a very narrow bandwidth. As revealed in [9], the large spring stiffness ratio is achieved by increasing the number of folded beams in series, whereby not only is a large area occupied, but the resonant frequency of the coupling spring also decreases, which may disturb the operation of the gyroscope, so a new kind of spring structure with a very small coupling stiffness is necessary.

### 2.2. Design of a Weak Coupling Spring System

**Figure 2.**
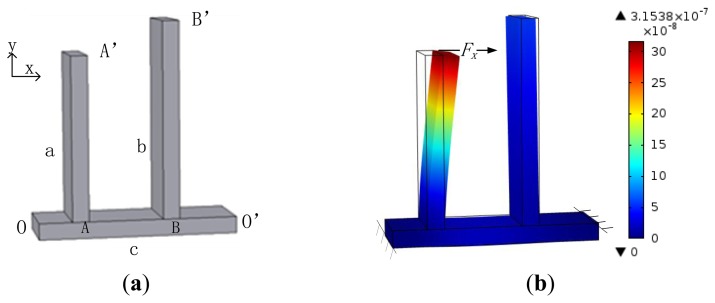
The weak coupling spring system. (**a**) The weak coupling spring system structure; (**b**) Deformation of the spring system when a force is applied.

The designed weak coupling spring system consists of three beams: two parallel beams a and b are fixed on a vertical beam c, both ends of c are fixed, as shown in Figure 2. Beams a and b are used as support springs, the beam c serves as the coupling spring.

To describe the mechanism of the spring system conveniently, two ends of the beam c are set as O and O′, the joint of beams a and c is A, the joint of beams b and c is B, the free end of the beam a is A′, the free end of the beam b is B′, as shown in Figure 2a.

The support spring stiffness is considered first. When a force *F_x_* is applied on one support spring such as the point of A′ of the beam a along the x axis, there is an angle between the line OA′ and the direction of the force *F_x_*, hence the united corner beam OA and a is rotated around O under the action of the torque $\vec{F_{x}}\times \vec{OA^{\prime }}$. Moreover, there is another torque $\vec{F_{x}}\times \vec{O^{\prime }A^{\prime }}$ that makes the united corner beam O′A rotate around O′. Under the action of the resultant moment, the beam c is bent, as well as the beam a. The net motion of A′ is dependent on the combined deformation of the bended beam c and the bended beam a, and the support spring stiffness of the beam a is the ratio of the force *F_x_* to the net motion of A′.

Considering the traditional coupling spring form that two support springs are connected by a coupling spring, as shown in Figure 1, if a force is applied on the joint of *k_c_* and *k_1_* along the x axis, *k_c_* is compressed much more than *k*_2_ due to its small stiffness, and the joint of *k_c_* and *k_2_* moves much less than the former joint. Considering the weak coupling spring system in Figure 2, when the beam c is bent, the beam b rotates around O′ along with the motion of B, and the net motion of B′ is very small. The deformation state of the weak coupling spring system under the action of a force *F_x_* along the x axis on the point A′ is simulated, as shown in Figure 2b. As revealed, the motion of A′ is far larger than the motion of B′, which indicates that the coupling spring stiffness is far smaller than the support spring stiffness.

On the basis of the above analysis, it can be inferred that the shorter the segment BO′ is, the less the motion of B′ is, and the smaller the coupling spring stiffness is. On the other hand, the decrease of the segment OA leads to the decrease of the resultant moment of $\vec{F_{x}}\times \vec{OA^{\prime }}$ and $\vec{F_{x}}\times \vec{O^{\prime }A^{\prime }}$, so the beam c is bent less, and the coupling spring stiffness is reduced. The resultant moment reaches max when A is in the middle of beam c.

### 2.3. Design of a MEMS Gyroscope with a Weak Coupling Spring System

**Figure 3.**
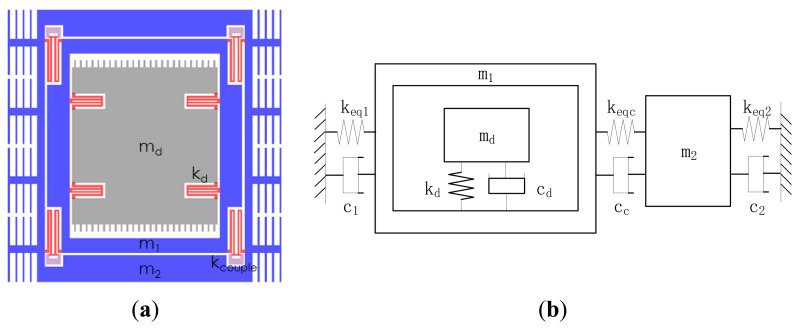
The MEMS gyroscope with a weak coupling spring system. (**a**) Schematic of the designed gyroscope; (**b**) Mechanical mode of the designed gyroscope.

The designed gyroscope is a single DOF vibration system in the drive direction, as well as a 2-DOF vibration system in the sense direction. The frame structure is used for minimizing the quadrature error, as shown in Figure 3. The drive mass *m_d_* is fixed on the active sense mass *m*_1_ by the spring *k*_d_, the passive sense mass *m*_2_ is out of *m*_1_ and connected to *m*_1_ with a weak coupling spring system K_couple_, of which the equivalent support spring stiffness is *k_eq_*_1_ and *k_eq_*_2_, equal coupling spring stiffness is *k_eqc_*. The mechanical model is established to describe to the vibration of masses, as shown in Figure 3b.

**Figure 4.**
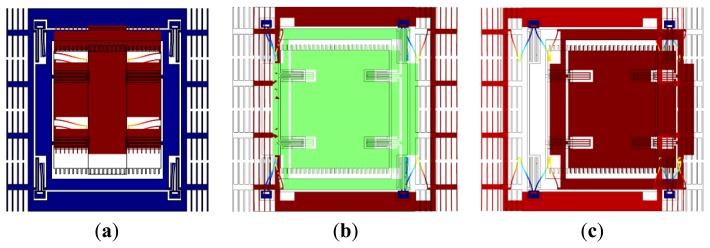
Vibration modes of the designed MEMS gyroscope. (**a**) Drive mode; (**b**) In-phase sense mode; (**c**) Anti-phase sense mode.

The drive mode is shown in Figure 4a, and the two sense modes are shown in Figure 4b,c. At the in-phase mode frequency, the masses move in phase, while at the anti-phase mode frequency, the masses move oppositely. The central frequency of the sense mode is in the middle of the in-phase mode frequency and the anti-phase mode frequency, near which the frequency response of *m*_2_ is flat. The gyroscope operates at the drive mode resonant frequency, which is designed at the central frequency, so the amplification factor changes little when the operation frequency drifts in a certain range [6].

Assuming the displacement of *m_d_* is *x_d_*, and the applied force on it is *F_d_*(*t*), the displacement Equation of *m_d_* along the drive direction is:

(7) $$m_{d}\;x_{d}^{\prime \prime }+c_{d}\;x_{d}^{\prime }+k_{d}\;x_{d}=F_{d}\left(t\right)$$

The displacement equations of *m*_1_ and *m*_2_ along the sense direction are:

(8) $$\left(m_{d}+m_{1}\right)x_{1}^{\prime \prime }+\left(c_{1}+c_{c}\right)x_{1}^{\prime }-c_{c}x_{2}^{\prime }+k_{eq1}x_{1}=k_{\text{eqc}}\left(x_{2}-x_{1}\right)+F_{c}\left(t\right)$$

(9) $$m_{2}x_{2}^{\prime \prime }+\left(c_{2}+c_{c}\right)x_{2}^{\prime }-c_{c}x_{1}^{\prime }+k_{eq2}x_{2}=k_{\text{eqc}}\left(x_{1}-x_{2}\right)$$

wherein, *x*_1_ represents the displacement of *m*_1_, *x*_2_ represents the displacement of *m*_2_, *F_c_(t)* is the Coriolis force sensed by *m_d_*, and can be expressed as *F_c_(t)* = *2m_d_ω_d_x_1_*′*(t)*, *ω_d_* is the resonant frequency of *m_d_*.

Based on the above analysis, the bandwidth minimum is only determined by the coupling spring stiffness in case of (*k_eq_*_1_ + *k_eqc_*)/(*m_d_* + *m*_1_) = (*k_eq2_* + *k_eqc_*)/*m*_2_ = *ω_s_^2^*. The ratio of *k_eqc_* to *k_eq_*_1_ is defined as *r_c_*_1_, the ratio of *k_eqc_* to *k_eq_*_2_ is defined as *r_c_*_2_. Equations (8) and (9) are rewritten in the frequency domain, as shown below:

(10) $$s^{2}\left(m_{1}+m_{d}\right)X_{1}\left(s\right)+s\left(c_{1}+c_{c}\right)X_{1}\left(s\right)-sc_{c}X_{2}\left(s\right)+k_{eq1}X_{1}\left(s\right)=k_{\text{eqc}}\left(X_{2}\left(s\right)-X_{1}\left(s\right)\right)+F_{c}\left(s\right)$$

(11) $$s^{2}m_{2}X_{2}\left(s\right)+s\left(c_{2}+c_{c}\right)X_{2}\left(s\right)-sc_{c}X_{1}\left(s\right)+k_{eq2}X_{2}\left(s\right)=k_{\text{eqc}}\left(X_{1}\left(s\right)-X_{2}\left(s\right)\right)$$

From Equation (11), the relation between *X*_1_(s) and *X*_2_(*s*) is derived and shown in Equation (12), and the transition function *H*_2_(*s*) of the displacement *X*_2_(*s*) against the Coriolis force *F_c_*(*s*) is expressed in Equation (13):

(12) $$X_{1}\left(s\right)=\frac{s^{2}m_{2}+s\left(c_{2}+c_{c}\right)+k_{eq2}+k_{\text{eqc}}}{k_{\text{eqc}}+sc_{c}}X_{2}\left(s\right)$$

(13) $$H_{2}\left(s\right)=\frac{k_{\text{eqc}}+sc_{c}}{m_{2}\left(m_{1}+m_{d}\right)}\frac{1}{\left(s^{2}+\frac{c_{1}+c_{c}}{m_{1}+m_{d}}s+{\omega_{s}}^{2}\right)\left(s^{2}+\frac{c_{2}+c_{c}}{m_{2}}s+{\omega_{s}}^{2}\right)-\frac{{\left(k_{\text{eqc}}+sc_{c}\right)}^{2}}{m_{2}\left(m_{1}+m_{d}\right)}}$$

When *s* = *jω*_0_ ≈ *jω_s_*, the gain of *x*_2_ is:

(14) $$H_{2}\left(j\omega_{s}\right)=\frac{k_{\text{eqc}}+j\omega_{s}c_{c}}{-\left(c_{1}+c_{c}\right)\left(c_{2}+c_{c}\right){\omega_{s}}^{2}-{\left(k_{\text{eqc}}+j\omega_{s}c_{c}\right)}^{2}}$$

Equation (14) reveals that if all the damping factors are zero, |*H*_2_(*jω_s_*)| is inversely proportional to *k_eqc_*, and if damping factors exits, |*H*_2_(*jω_s_*)| increases also along with the decrease of *k_eqc_*. According to the above analysis, it can be inferred that the designed gyroscope with the weak spring system has optimized the amplification factor of the sense mode.

## 3. Results and Discussion

**Figure 5.**
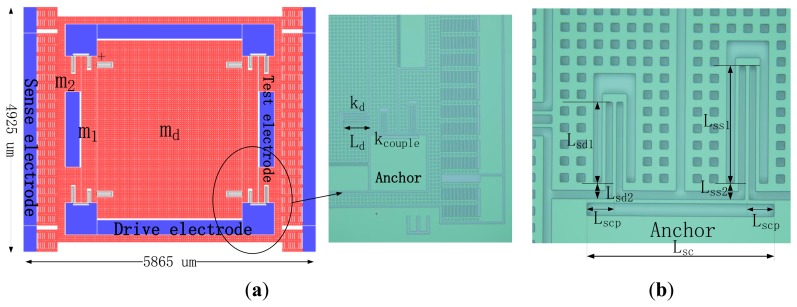
Layout of the designed gyroscope. (**a**) Layout and partial photo of the designed gyroscope; (**b**) Photo of the designed weak coupling spring system.

The layout and a fabricated gyroscope are shown in Figure 5, wherein interdigitated comb-drivers are used to drive the gyroscope, groups of parallel-plate capacitors serves as the sense structure, the quadrature adjustment electrodes are on sides of the sense electrodes, and extra test electrodes are placed to sense the motion of *m*_1_.

Each support spring of *m_d_* consists of four folded beams to achieve good linearity, while each support spring of *m*_1_ or *m*_2_ consists of three folded beams, they are joined on a coupling spring as shown in Figure 5b. Since the coupling spring stiffness is dependent on the distances between anchors and joints, the springs of three folded beams are capable of having joints nearer to the anchors with less fixed area.

The proposed gyroscope is fabricated on a n-type (100) silicon on insulator (SOI) wafer, with a thickness of 20 μm, the designed width of all beams are 10 μm, and all holes on the structure are 20 × 20 μm^2^. The other parameters are shown in Table 1.

**Figure 6.**
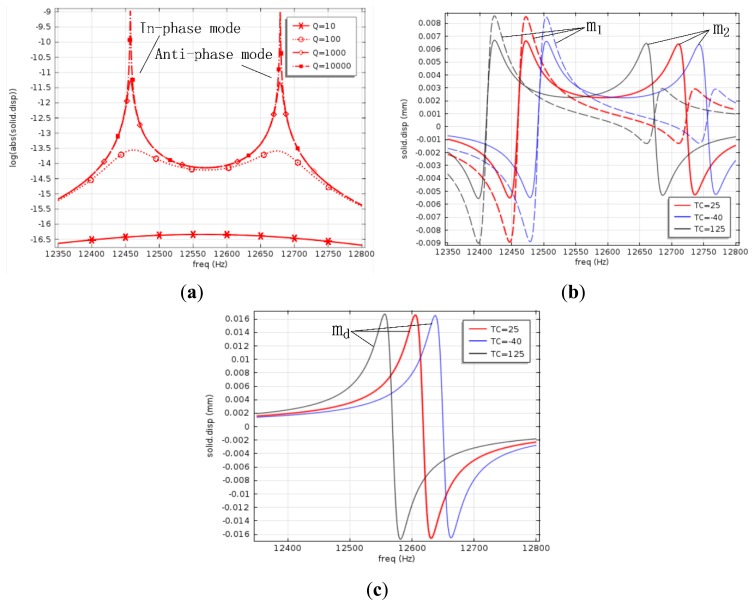
Simulated frequency response of the designed MEMS gyroscope. (**a**) Frequency response of *m*_2_ against damping factors at room temperature; (**b**) The sense mode frequency response against temperature variations; (**c**) The drive mode frequency response of against temperature variations.

The gyroscope structure was fabricated using ICP etching, before which Al was ion beam sputtering deposited as etching mask. After the Al was corroded, the movable structure was released by etching sacrificial silicon dioxide in 49% aqueous HF solutions at 60 °C. The release process takes about 280 s. The central frequency amplification factor of the 2-DOF vibration system shown in Figure 1 is determined by damping factors as well as the coupling spring stiffness. The effect of damping factors is considered here first. Since anchor loss is the main energy loss mechanism when the gyroscope operates in high vacuum, the impact of *c_c_* can be ignored [12]. In order to make the simulation simple, the quality factors of each spring mass system are assumed to be equal, and their value is Q. The frequency response of the passive mass *m*_2_ against damping factors is simulated, as shown in Figure 6a, which indicates that when Q changes above 1,000, the central frequency amplification factor is invariant. Quality factors of most MEMS gyroscopes are over 1,000, so the designed gyroscope is insensitive to damping factor variations, and it is reasonable to use 1,000 as the value of Q in the next simulations.

In Figure 6b, the solid lines are displacement-frequency response curves of *m*_2_, dotted lines are displacement-frequency response curves of *m*_1_ in the sense direction. At room temperature, the central frequency is 12.59 kHz, the frequencies of two modes are 12.47 kHz and 12.71 kHz, the peak to peak bandwidth is about 240 Hz, and the amplification factor at the central frequency is 91, which reveals a good tradeoff between the bandwidth and the amplification factor.

Both frequency responses of the drive mode and the sense mode are sensitive to the dimensional variations due to fabrication tolerances, and the resonant frequency separation induced by stable process is usually less than tens of Hertz [7]. Simulation results in Figure 6b reveal that the amplification factor varies up to 10% when the operation frequency drifts from 12.55 kHz to 12.63 kHz at room temperature, having a flat range as wide as 80 Hz, so even if there is a frequency separation caused by process variations, the operation frequency is still located in the flat region, which indicates that the amplification factor of the designed 2-DOF gyroscope is insensitive to fabrication errors.

Young's modulus *E* and Poisson's ratio *ν* of silicon are dependent on crystal directions as well as the temperature, and the temperature coefficient of *E* for the [110] direction is −131 × 10^−6^ °C at room temperature [13], so the frequency response varies with temperature. The displacement-frequency response of *m*_d_ against temperature is simulated and shown in Figure 6c, and that of *m*_2_ and *m*_1_ is shown in Figure 6(b), wherein red lines represent the frequency response at 25 °C, gray lines represent the frequency response at 125 °C, and blue lines represent the frequency response at −40 °C. It can be seen that the resonant frequency of *m*_d_ varies 3 Hz from −40 °C to 25 °C, and 5 Hz from 25 °C to 125 °C, while the central frequency of *m*_2_ varies 6 Hz from −40 °C to 25 °C, and 10 Hz from 25 °C to 125 °C, revealing that the resonant frequency of *m*_d_ is always in the flat region of the frequency response of *m*_2_ within the whole temperature range. Therefore, the designed 2-DOF gyroscope is insensitive to temperature variations.

The frequency response of the 2-DOF vibration system in the designed MEMS gyroscope was tested with a Scanning Laser Doppler Vibrometer. The gyroscope was placed on a PZT in a sealed tank with the air pressure 6.7 Pa at about 22 °C in test, an AC exciting force is applied on the substrate side of the gyroscope along the sense direction, the force is provided by a PZT exited by a group of periodical chirp electric signals, and the vibration signal of microstructure was acquired by the Scanning Laser Doppler Vibrometer.

**Figure 7.**
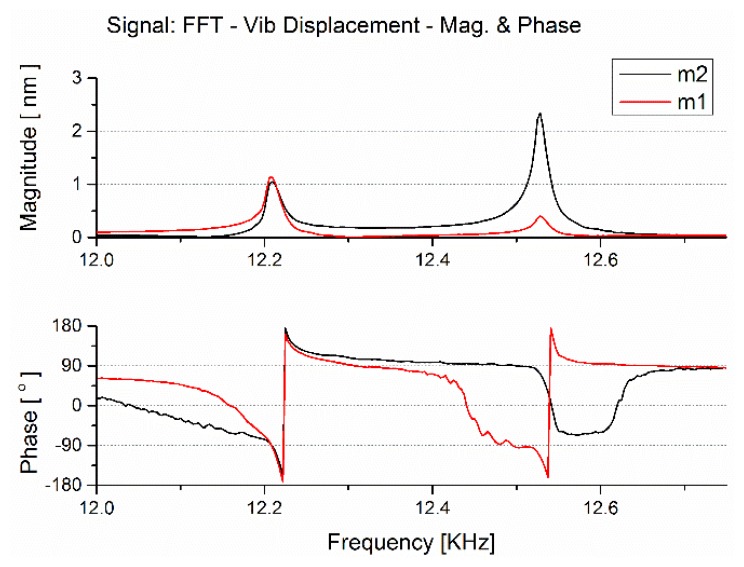
The tested sense mode frequency response of the fabricated MEMS gyroscope.

The amplitude-frequency and phase- frequency response of different masses is shown in Figure 7, gray lines represent the frequency response of the mass *m*_2_, and red lines represent the frequency response of the mass *m*_2_. Due to fabrication errors, the measured central frequency is about 200 Hz below the simulation, and the tested peak to peak bandwidth is about 284 Hz, both are still in agreement with the simulation result capable of meeting the needs of practical application. The vibration magnitude at the central frequency 12.302 kHz is 0.384 nm, and the magnitude far from resonant frequencies is about 9 pm, from which the amplification factor at the central frequency is about 42.7, not as good as the simulation value, which is due to different stimulation ways on masses between in test and in simulation, but the result is still encouraging. The flat range is 118 Hz when the magnitude varies 10% compared to the magnitude at the central frequency.

Compared to the former multi-DOF gyroscopes described in [6–11], the designed MEMS gyroscope with the weak coupling spring system is capable of operating at higher frequency, having narrower bandwidth as well as larger amplification factor, and shows the potential to make a better tradeoff between the amplification factor and the bandwidth. Besides, the size design is more flexible, without extreme sizes to realize narrow bandwidth.

## 4. Conclusions

The performance of a MEMS gyroscope is influenced a lot by the mechanical impedance, and due to the decrease of the amplification factor, the mechanical impedance of gyroscopes with 2-DOF sense mode is increased compared with that of gyroscopes with single DOF sense mode, so the sensitivity and the bias stability of a multi-DOF MEMS gyroscope is not that good in theory, but the robustness is attractive. Still there are ways to improve the performance of multi-DOF MEMS gyroscope, like improving the quality factor of the drive mode, or enlarging the amplification factor of the sense mode.

The 2-DOF vibration model used in this paper has parameters adjusted more conveniently to make a tradeoff between the bandwidth and the amplification factor. Besides, a weak coupling spring system of tiny stiffness is proposed to achieve a narrow bandwidth, and the designed gyroscope with the weak coupling spring system has an amplification factor of 91 at with the central frequency, proving good robustness while no sacrificing sensitivity.

## Figures and Tables

**Table 1.** Key parameters of the designed gyroscope.

**Designed Parameters**	**Value**	**Designed Parameters**	**Value**
L_d_ (μm)	291.5	m_d_ (mg)	0.232
L_ss1_ (μm)	279	m_1_ (mg)	0.122
L_ss2_ (μm)	40	m_2_ (mg)	0.198
L_sd1_ (μm)	192	K_d_ (KN/m)	1.49
L_sd2_ (μm)	40	K_eq1_ (KN/m)	1.26
L_sc_ (μm)	340	K_eq2_ (KN/m)	2.27
L_scp_ (μm)	10	K_eqc_ (KN/m)	0.056
